# The emerging radiographer’s transient community service professional career pathway

**DOI:** 10.4102/hsag.v24i0.1280

**Published:** 2019-10-17

**Authors:** Bonita B. Johnson, Chandra R. Makanjee, Willem A. Hoffmann

**Affiliations:** 1 Department of Biomedical Sciences, Faculty of Science, Tshwane University of Technology, Pretoria, South Africa; 2 Department of Medical Radiation Sciences, School of Clinical Sciences, Faculty of Health, University of Canberra, Canberra, Australia

**Keywords:** community service year, community service radiographer, student radiographer, graduate radiographer, professional role, community service placement site, transition, professional role, expectations, experiences

## Abstract

**Background:**

Post-qualification regulatory 12-month community service (CS) was implemented in South Africa in 1998. Since the implementation, studies have been conducted in various disciplines to measure the impact on health services and on the affected professionals, but these did not include radiography professionals.

**Aim:**

This study explored the expectations and experiences of student radiographers in respect of the CS concept as an integral transitional career pathway from the student radiographer role to that of a provisional practitioner in transit to acquiring registered radiographer practitioner status.

**Research methods:**

The research design entailed a qualitative exploratory approach using a longitudinal data collection approach. That is, data collection from the purposefully selected student radiographers’ focus group discussions, as well as from placement institutions’ qualified professionals and managers, formed the triangulated data sources. In addition, individual interviews were conducted post-placement until data and thematic saturation had been reached. Tesch’s (1990) method was used for the data interpretation and analysis.

**Results:**

The themes that emerged reflected the preparedness of these students to fulfil the requirements, their experiences of their anticipated placement institution, preparedness for their roles and responsibilities and uncertainties about their readiness for the actual encounter. A golden thread throughout was critical self-reflection on their ability, adaptability and capability to meet the requirements of the system, namely the community placement institutions and appeasing the Department of Health.

**Conclusion:**

The study illustrates, by means of a framework, the student radiographers’ journey in transit to acquiring eligibility as registered radiography practitioners in a regulated career pathway.

## Introduction

Given the contemporary evolving nature of imaging technology that impacts the way diagnostic imaging services are delivered, as well as the increased global demand for these imaging services, coping with the demand supply chain involves the upkeep of radiography practice (Sloane & Miller 2017). Inherent is an ongoing challenge of a critical needs analysis and undertaking a wide review of existing services.

In the African context, constraints are faced in retaining skilled, experienced professionals to deliver a timely equitable service, apart from financial constraints in sustaining healthcare services (Hatcher et al. 2014; Ngoya, Muhogora & Pitcher 2016). In South Africa, a regulatory requirement of a 1-year period of community service (CS) for health professionals was implemented in 1998 for medical providers (Bhengu 2006) and in 2003 for allied healthcare professionals. That entails an allocated 12-month period of mandatory service in public institutions on completion of their formal training, in a CS provisional practitioner role. Thereafter, they become eligible to register with the Health Professions Council of South Africa as a professional practitioner. The CS programme is intended to ensure equity of access to all health services’ users and secondarily to give novice professionals the opportunity to develop skills and to acquire knowledge, behaviour patterns and critical thinking, resulting in professional development (Reid 2002; Roziers, Kyriacos & Ramugondo 2014).

Since the implementation, numerous studies have been conducted involving a diverse range of healthcare professionals, but only one national study by Hatcher et al. (2014) mentioned radiographers as part of the background. A recent study by Dlamini, Sekholi and Bresser (2018) explored experiences of post-community placement. A brief literature overview on CS as an explicit requirement for registration as a radiography practitioner was undertaken internationally. Several studies focussed on helping healthcare professional learners to develop through targeted community engagement in preparation for providing healthcare to the broader community (Fisher et al. 2018). No studies were conducted in the context of a national regulation to undertake mandatory CS to reach registered practitioner status as an intervention to bridge inequality in health service delivery. In Australia and New Zealand, some tertiary institutions currently have a professional development programme requirement for radiographers with provisional registration with the Australian Health Practitioner Regulation Agency and New Zealand Institute for Medical Radiological Technologists, to undertake supervised practice should they not have the requisite capabilities as professional health practitioners (Australian Health Practitioner Regulation Agency 2016; Medical Radiation Practice Board of Australia 2014:3).

Globally, workplace learning in a radiography programme provides students opportunities to learn and engage in a real-world environment. The emphasis is on acquiring skills and competencies in conducting imaging investigations, engaging in administrative processes, a research component and a residency or an internship to prepare graduates for the workplace environment. An additional benefit is enhanced student awareness of appropriate professional behaviour in the workplace, including skills development in areas such as communication and interpersonal relationships (Fisher et al. 2018).

## Aim

The aim of this study was to generate insights into emerging radiography professionals undertaking three transient professional role changes as they journeyed through their CS from pre- to post-placement, and additionally to explore community placement site professionals’ expectations and experiences of these emerging professionals transitioning to acquiring a registered professional radiographer practitioner.

## Methods

### Study design

An inductive exploratory research design with a longitudinal component was employed to collect rich data by means of focus group (FG) and individual interviews conducted on two occasions (the first in August and September 2010 of the third year and the second 12 months later). This enabled gaining insights into participants’ understanding and meaning making whilst undertaking the temporary transitional career journey and undergoing change in professional roles and identity (Rubin & Babbie 2016).

## Setting

All four academic institutions of higher learning offering radiography in the Gauteng province were approached to participate in the study. Only three of the four academic institutions agreed to participate in this study. The three public CS placement centres, which are also accredited training sites for these academic institutions, located in the same province, agreed to participate, of which two were tertiary healthcare institutions. All of these training sites provide a diverse range of medical imaging services. These institutions were selected for ease of access to obtain the required data, based on the explorative nature of the study design and the high probability of accessing CS radiographers from other provinces as well. At these institutions, suitable locations were identified in liaison with their heads of department to conduct the interviews with minimal disruption of radiological services.

## Study population

The study population comprised final-year diploma and Bachelor of Radiography students (*n* = 17) who agreed to participate in both the FG discussions (FGDs) (*n* = 3, five to six participants per group; FG 1: two male participants and four female participants; FG 2: six female participants; FG 3: five female participants) and post-placement individual interviews. However, only nine (seven females, face-to-face; two males, telephonic) were available for these interviews. This was followed by FGDs (*n* = 3, three to six per group: female participants) involving junior, senior and chief radiographers. The last group comprised clinical heads of radiography (*n* = 3 females) of the three community placement centres who were individually interviewed.

## Sampling

Sampling for the final-year radiography students entailed purposeful sampling strategy from the three academic institutions, whilst the junior, senior and chief radiographers were by means of convenience sampling from the three community training centres. Lastly, the clinical heads of radiography of the three community placement centres were purposefully sampled.

## The instruments

Data collection was entailed by use of semi-structured interview guides. Some key questions were formulated to assist in exploring the concepts at hand. However, participants were also allowed to diverge to pursue an idea or response in more detail. According to Gill (2008:291), this is useful for the discovery or elaboration of information that is important to participants, but may not have previously been thought of as pertinent by the research team. Prior to their placement, the interview with the student radiographers commenced with an icebreaker: ‘What is your understanding of CS?’ followed by ‘Can you share the expectations you have regarding the upcoming CS year?’ Depending on the responses, issues of placement were probed further.

The healthcare radiography professionals’ interview guidelines were informed by preliminary findings from the FGDs with student radiographers, as was the interview guide for the heads of radiography. The interviews commenced with probes included: ‘do the CS radiographers meet your expectations?’, ‘Share some experiences of the CS radiographer encounters from a supervisory level’. The responses were checked for similarities from the previous preliminary data interpreted and analysed and if new information was shared, it was followed with probes. The duration of the interviews was approximately 15 min to 1 h and 20 min.

Lastly, the actual CS experiences were captured after completion of the service year to establish whether the pre-existing expectations and experiences were realistic. Probes entailed: ‘Can you please share the experiences of the community service placements?’, ‘Were your expectations met as discussed in the focus group discussions?’ Depending on the responses, the participants were probed further until data saturation. The duration of these interviews was approximately 45 min.

## Data collection

Prior to data collection at the various research sites, information sessions were held with students separately from the healthcare radiography professionals and their respective supervisors. These sessions were used among others to inform them on aspects of privacy and confidentiality and the voluntary nature of participation. This was followed by obtaining consent, and only participants who agreed to take part were included in the study.

**FIGURE 1:**
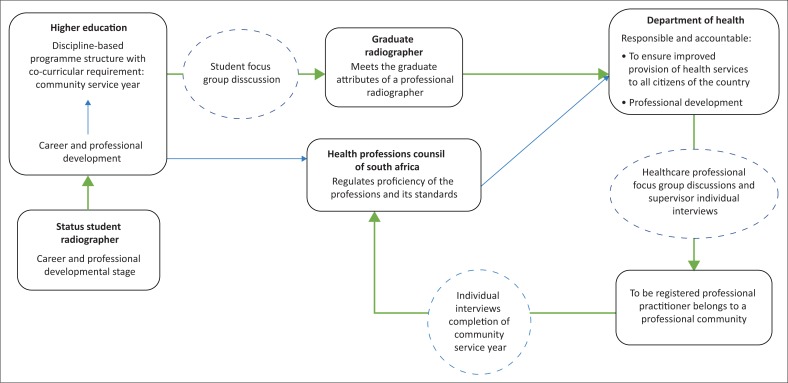
The various data collection points.

The actual data collection (see Figure 1) commenced with the student radiographer FGDs prior to placement. The preliminary data interpretation and analysis directed the subsequent FGDs with healthcare radiography professionals and individual interviews with their respective heads of department.

Lastly, post-placement individual interviews were conducted to capture rich data prior to the participants’ registration with the Health Professions Council of South Africa as practitioner.

## Data analysis

The digitally captured data were supported by field notes and verbatim transcriptions by the first two authors. The first and third authors interpreted and analysed the data, which entailed identifying preliminary codes and categories. These were used to inform on the subsequent interviews to follow until data saturation was reached (Tesch 1990:138). Similarities and differences of emergent themes moulded the phase of the interviews that followed. After completion of all these interviews, the interpretation and analysis process followed through re-alignment with the previous emerging themes and subthemes, until thematic saturation was reached.

## Trustworthiness

The first two authors firstly performed independent analysis of the data; thereafter, all three researchers contributed to the refinement of themes and interpretations (Green & Thorogood 2009). The first two authors who performed the data analysis are experts in the field of radiography and have substantive knowledge and experience in clinical practice and community engagement. None of the authors was part of the final-year radiography teaching team. To note, the third author was not a radiography field expert but an expert in the psychology. The second and third authors are also experts in qualitative research.

The FGs’ interviews were audiotaped and transcribed. Per verbatim quotes of participants were used. Member checking was performed by compiling a draft of the preliminary categories, and themes including the supporting quotes were sent for comments to all the participants. Comments from the participants were then integrated (Schoot et al. 2005).

Trustworthiness was further enhanced by means of an audit trail and triangulation of data sources obtained from student radiographers, supervisors and managers and professional radiographers (Welman, Kruger & Mitchell 2010).

### Ethical consideration

Ethical clearance to conduct this study was obtained from the Tshwane University of Technology Research Ethics Committee (Ref#: 2010/07/006). Permission and approval were obtained from three of the four academic training and CS healthcare placement sites in the Gauteng region. The research participants’ anonymity was assured and protected by assigning codes to the respective FGDs and pseudonyms to participating institutions. Only the researchers had access to the raw data captured. All digital data were password-protected (Denzin & Lincoln 2005).

## Findings

The findings are structured according to the mapped career pathways, namely, providing perspectives from a student role, that is, preparation for transitioning to the CS role, the journey through actual CS encounter and post-placement and, lastly, the community placement site perspectives on CS. Only the most significant findings, with quotations, are discussed with the literature support. Throughout the findings, the codes are, for example, FG1 – ‘Focus Group’ and number indicates first (1); I4 – “Individual interview” of participant 4; MFG1 – ‘Mixed Focus Group’ and the number indicates first (1).

### Preparation and readiness for transitioning to community service role

Transitioning to a professional practitioner role is an inherently stressful experience (Laschinger et al. 2016). This transition encompasses the intermediate role of a provisional practitioner required to deliver CS. This preparatory phase requires the ability to make the correct decisions and course of action to function optimally. Preparation and support are important to ensure a smooth transition.

### Dissemination of information and strategies to fill information gaps

During the FG interviews, views were shared on the adequacy of relaying information on what the processes entailed, for instance:

‘There was a sheet that explained when you’re going to get the feedback with dates telling when they [*are*] going to do this, …the meeting, … then eventually, … the 22nd September, … it’s going to be out … about the year, … nothing about that.’ (FG3, P4, female, student radiographer)

However, questions about the meeting and salary were answered:

‘[*The guardian lecturer*] and [*student B*] went to a meeting … they got the information. … it was a broad thing, but it wasn’t complete. So, we knew that was the salary, … if you work at a rural place, you get rural allowance. … If you are not working in a rural area you just get a basic salary.’ (FG2, P4, female, student radiographer)

To fill these information gaps, a few participants explored: ‘… asked people [*radiographers*] questions who went to CS. Some people told me, this place is nice, that place is nice, don’t go there’ (FG2, P1, female, student radiographer). A few spoke to advisors:

‘… [*T*]he head of department … I asked her, ‘what I must do? … she said ‘just put down [*CS health care facility A*] as your first choice and your only choice’. So, they can actually see from that that you only want to stay there …’ (FG3, P3, female, student radiographer)

The information gap that was not filled because of a co-curricular requisite was, ‘… what you have to do in the department and that we are going to be assessed yearly and I don’t know how often’ (FG3, P1, female, student radiographer).

### Unpredictable placement and practicalities

During the FGDs, students spoke about navigating practicalities, such as relocation and the impact this might have on not being adequately prepared (Purnell & Hoban 2014). Firstly:

‘It makes you wonder, now you’re stressed again, did they give [*the guardian lecturer*] a notice that says everyone got their first choices now or are you not in the second round. Then hours after that OK, these people are again in the second round.*’* (FG2, P2, female, student radiographer)

Decision-making on the choice of placement was also affected by uncertainties:

‘So that you know even beforehand, before choosing a CS centre to do your CS … so if I am going to adjust working throughout, you know you have to mentally prepare yourself as well …’ (FG2, P6, female, student radiographer)

‘… [*L*]ike it is going to be a one bedroom with a kitchen, … rooms. … are you sharing or what is happening? … It is said that accommodation is provided, but they don’t mention. … It’s like a whole lot of hassle …’ (FG2, P5, female, student radiographer)

This was overcome by being proactive, for instance:

‘It was … a worry because we arranged, … phoned the people … the one said, ‘No we are not going to stay there, they’re still arranging accommodation’. The other one said, ‘No that’s not true it’s already been arranged’. So … we didn’t really know what was going on. …the CEO told us, that we are staying there. So, then we were a little bit more settled …’ (I4, community service radiographer)

The new role is not just an obligatory requirement but opportunities for lifelong learning, professional development: most participants were positive and motivated: ‘… it’s a great opportunity for us’ (FG3, P5, female, student radiographer). This was illustrated in values, social responsibility and accountability for contributing:

‘… [*W*]e must not even forget that as a student we used to practise with the patient and now it was the time that we must just give back to that community, to the country as well.’ (FG4, P2, female, community service radiographer)

Examples included awareness in terms of prioritising professional gain, values, culture and ethical conduct versus monetary gain (Billet & Somerville 2004; Jackson 2016):

‘… [*G*]o work there, help some people. … learn myself too and grow. So, I would not [mind to] be placed in a not so nice place. So if you work hard and achieve your goals you can also be better than that.’ (FG3, P2, female, student radiographer)

‘I also think the money isn’t the main perception in this case, … I’ll get to share some stuff with them or support them …’ (FG1, P6, male, student radiographer)

## The actual community service placement encounter

Transformative learning is central to critical incident learning, which involves finding potential solutions to challenges and dilemmas in workplace scenarios (Jackson 2016). Trede, Macklin and Bridges (2012:374) state that it is ‘A way of being and a lens to evaluate, learn and make sense of practice’.

### ‘You will always have something you won’t like at your workplace’

According to Jackson (2016), the workplace incorporates dimensions of life skills, career management and personal circumstances. Non-technical skills are critical, such as teamwork, communication, critical thinking and self-management, with the focus on exploring the ability to perform, for example:

‘… [*W*]e were three radiographers … one had to go to theatre, one doing scans, I was left alone on the floor. … there were literally 30 people waiting to be helped on trolleys bleeding. …. She (the head radiographer) told me to cover screening as well as portables. … I told her, ‘I can’t cover portables as well’. ‘Can’t you see it’s busy’, she started screaming at me.…‘I’m not willing to assist my peers’. I started crying … I didn’t know what to do.’ (I1, female, community service radiographer)

Of extreme importance in any workplace or community context, is the people with whom one interacts and the relational aspects (Eraut 2000), as illustrated in these quotations:

‘There was a time in the middle of the year … I felt that I can’t do radiography anymore. As a student, I loved radiography. …In the middle of the year, I hit it like rock bottom. It’s like I hate this, I just want to do something else. I applied for radiation therapy (laugh). But later … I’m like, I’m starting to enjoy it again. I think I’m learning to cope with the stress that’s been placed on me. … I’m just starting to, like in the middle of the year, I would just do a patient and not speak to them or anything. … Now I’m … spending more time with my patients, staying calm, being friendly and I’m actually enjoying it once again.’ (I1, female, community service radiographer)

On the contrary:

‘I am feeling quite confident choosing the job that I did. … I communicated more with the patients … In the private sector you can’t always share something with the patient. It’s just work, work, … In the (CS placement site), a smaller hospital, fewer patients you can build a relationship with the patient, encourage … and help them. … I’m actually staying on … next year. … you will always have something you won’t like at your workplace, unfortunately. … I kind of like the people I am working with and our supervisor is also very nice, she looks after you.’ (I6, female, community service radiographer)

### Varied skills enrichment opportunities with complex judgemental calls and decisions

Placement sites varied in terms of spaces for refining their skills (Richard et al. 2016), participants were able to reflect on their competencies. For example: ‘my radiography skills have improved; my theatre skills have improved. … I can do a skull on a drunk guy in any position now’ (I1, female, community service radiographer). Problem-solving and critical thinking were applied when equipment was not functioning optimally:

‘I think I most definitely improved (my skills) on the normal radiography x-ray machines. The basic stuff … to improvise if the machine cannot move all the way …’ (I6, female, community service radiographer)

At placement sites undergoing renovations, in the absence of opportunities for hands-on practice, the results were less positive:

‘If, now, I had to do a CT scan, I wouldn’t know where to start … and screening and other things … it’s a disadvantage. I didn’t get a chance to fully practise what I was taught at (university). …Now … already a year passed without practising …’ (I8, female, community service radiographer)

Some of the realities to deal with were structural protocols and procedures. In a few instances, participants were inducted appropriately, very similar to the controlled setting (Sen Gupta et al. 2014):

‘When I came, they gave me a form that I had to fill in. That form stated what is my role in the department, what I must do and how I must treat my patients. They also gave me another orientation around the machines, the hospital itself and the procedures that are done here.’ (I8, female, community service radiographer)

In other instances, participants had to deal with a mismatch of role expectations between the mature professional versus the novice when making judgement calls and decisions. Ortiz (2016) describes this phase as being in the process of acquiring professional confidence to provide safe, competent care. This inadequacy resulted in feeling:

‘Overwhelmed, freaking out. We asked the people, what’s the protocol? What to do? They were like, ‘Just do the x-rays!’ We don’t know which views we had to do, where to start. They showed us the rooms and that’s about it.’ (I1, female, community service radiographer)

The absence of adequate support resulted in confusion on what they could do and what not:

‘… We got in a lot of trouble for doing oblique views and … an open mouth for a c-spine … lateral chests … the head radiographer tells us the doctors don’t know what they’re doing.’ (I1, female, community service radiographer)

### The administrative processes and procedures for professional competency appraisal to practitioner eligibility and registration

As part of satisfying standards, the obligatory CS requirement was also the ‘performance appraisal’, a set of criteria prescribed by the Department of Health (2007:1) to submit to the Health Professionals Council of South Africa’s Radiography and Clinical Technology Board to acquire registered practitioner status. A few experienced the process as applied fairly:

‘Yes, he [*the manager*] did explain to me that every quarter he will sign a report about my conduct,… my work assessment, how I performed … And it did happen like that every four weeks … The final report, around December, I signed it, that I’m finished with my CS …’ (I8, female, community service radiographer)

However, most experienced the process as inconsistent, and one participant shared an experience of having to make a moral judgement and a decision not to submit to this:

‘The head radiographer came to me literally like a month ago [*October*]. She said that she has to evaluate us or give us marks every four months or something. She’s like, ‘Just sign all these forms and I’ll fill it in later’… I complained and I refused to sign it.’ (I1, female, community service radiographer)

## Perspectives on community placement site expectations and experiences

Challenges were experienced in addition to providing an equitable service to everyone accessing these services.

### The transient challenged experiences

In the process of transitioning, both CS placement sites and the learners shared their uncertainties at the initial phase of placements issues, of issues that they had little control over (Budhai & Grant 2018).

During the FGDs, it became obvious that CS placements ‘add to the value that we have in government’ (MFG2, P2, female, senior radiographer). Upon entering the workplace environment, they found that the reality was ‘shortage of skills’ (MFG2, P2, female, senior radiographer) and ‘no staff, the small hospitals are understaffed’ (MFG1, P6, female, senior radiographer). In some instances, the placement was viewed as a temporary source of relief: ‘after the year you go back to your problem of short staff; it’s like even if they promise you five radiographers, you get three. Because they were allowed to swop’ (MFG2, P1, female, chief radiographer). Then commencement of these services was delayed, as stated:

‘They promised them accommodation, at the end of the day, you do not get it or you get it like now, March. Some of these CS radiographers come from distant areas and it is too far for them.’ (MFG2, P1, female, chief radiographer)

The CS service site radiographers were in support of novice professionals being assigned to their preferred placement location. But the reality was:

‘(Students) should choose where they want to go, the thing is they [*Department of Health*] should honour that. The problem is they [*Department of Health*] place them, then change their mind. I think they (students) should choose where they want to go but they should not be able to change it.’ (MFG2, P1, female, senior radiographer)

### Facilitative role in transitioning the novice professional undertaking community service

These radiographers, apart from their traditional supervisory role, had to enable the smooth progression of a gradual transitioning to be proficient in practice by means of formal supervision:

‘… The first two months, you are under strict supervision, then you get to experience the workstations all alone. If you have problems, there are people you can ask for help …’ (MFG3, P2, female, senior radiographer)

This transitioning was also facilitated by ‘… teach[*ing*] them what to do and what not to do’ (MFG2, P2, female, chief radiographer) because of a mismatch, ‘Like they are still students, we expected more’ (MFG3, P2, female, senior radiographer).

The disruption is highlighted:

‘… [*I*]f you come in your first week (and you are) doing scans, you do not know anything, you are actually holding the people back. They need to teach you first before they put you there.’ (MFG1, P4, female, senior radiographer)

### ‘Know the job and just get in and move on’ (MFG2, P1, female, senior radiographer)

In terms of professional competency, proficiency was judged by using the basic skills with minor adaptations in performing their daily tasks and the ability to make a judgement call and a decision:

‘… Radiography-wise everything is the same. It is just this hospital the big thing it’s digital. If you come from cassettes and you are coming to ours it is way different … and the last thing is that you must make a decision on your own now … It is not like you when you were a student and you confirmed with somebody, you have to know that this is right, and wrong.’ (MFG1, P5, female, junior radiographer)

This was highly dependent on being acquainted with the inherent role and responsibilities of a professional practitioner; issues of personal and professional attitude and willingness play a role in an enabling journey:

‘To me radiography is constant. It does not matter where you work, there are certain things that you need to do and need to know to be a radiographer. … it is on a personal level. … How quickly you want to learn … adapt to this environment.’ (MFG1, P3, female, junior radiographer)

It also depended on the placement preference, which could result in undertaking a CS year at the accredited training site. This was an advantage in terms of transitioning into the environment:

‘It is quite different when you get people from other learning institutions because, students from institution C, it is very easy when they come to CS healthcare facility VII. We trained here as students, we are familiar with the place, when somebody else comes from another institution, they have to still adapt to the place and the protocols.’ (MFG3, P2, female, senior radiographer)

### Staying up to date with the acquired skill and professional growth

A challenge was to retain skills that are highly dependent on the CS site operational structure, whether fixed or flexible. Attitude also plays a role in venturing out of one’s ‘comfort zone’:

‘If you want to grow, you have to go outside and see what’s happening there. I mean you studied here and then you do your CS (at another place) …’ (MFG1, P6, female, senior radiographer)

‘It depends on the hospital. … Here you do not rotate, you either work in general radiography or you work in CT [*computed tomography*] scan or…MRI [*magnetic resonance imaging*] … At the other hospitals … [*you*] rotate …’ (MFG1, P4, female, junior radiographer)

### Professionalism and cooperative behaviour

Community service placement radiographers appraised the novice practitioners for their motivated effort to utilise opportunities in refining their knowledge and skills by actively engaging with their seniors:

‘… [*T*]hey still listen to us, we can give them work to do and they … do it. They ask us questions, they come to us when they do not know anything or when they want to learn … So, they do see us as their seniors.’ (MFG3, P1, female, chief radiographer)

## Discussion

**FIGURE 2:**
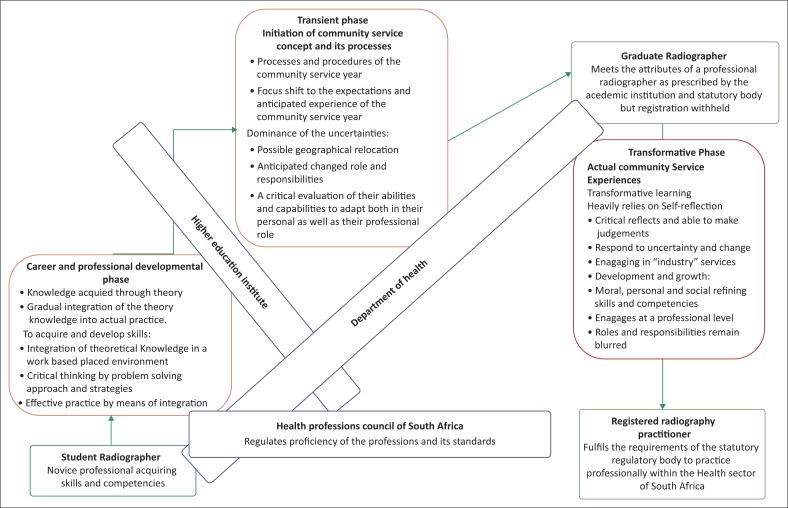
Framework illustrating the various stages and between spaces in the transient and transformational roles to become a registered professional practitioner.

The structure of the discussion is guided by a framework (see Figure 2). In a learner role, the career and professional development phase and service learning to acquire profession-specific clinical knowledge, skills and abilities (Keen & Pease 2016; Qenani, MacDougall & Sexton 2014) are dominant. The focus on complying with National Higher Education and Health Professional Council statutory body requirements (Ness et al. 2016) shifts towards the Department of Health requirement. Although essential to their future health professional roles, in the ‘community context’, the value shifts to acquiring a broader, more generic skillset, such as ‘life-wide learning’ with regard to the ‘CS’ role (Thompson et al. 2013).

Based on the findings, participants clearly understood the concept and value of CS and its envisaged outcomes (Paterson, Green & Maunder 2007). In the initial intermediate transient phase, the novice professional is trying to make sense of the anticipated changed professional identity and at the same token deal with the ‘blurred’ role and responsibilities (Ryan & Carmichael 2016), finding themselves ‘in the between spaces’ (see Figure 2). In this space from the student to the provisional practitioner role, the expectation was in a short space of time to make a timely, but also an effective decision on a CS placement site, which may not be successful (Laschinger et al. 2016) and thus making these professionals vulnerable. In addition, the transient phase represents a changing role within the ambit of a ‘CS radiographer’, yet a novice professional faced with the real workplace environment and its characteristics. These disruptions affected their lives both personally and professionally, making them more vulnerable. This leads to emotional distress, a sense of insecurity, self-doubt and critical reflection on their abilities, capabilities and confidence (Duchscher 2009; Parker et al. 2014). The response to the question, ‘How to manage this transition?’ led to taking a proactive approach by visiting the prospective sites to have face-to-face conversations with radiographers at the placement site. However, their anticipated role and responsibilities in most instances were not addressed. This is an indication of building confidence and motivation whilst still honing their professional ability and capabilities (Fisher et al. 2018).

Regarding the actual service placement, the transformative phase (see Figure 2), understanding of role transitions and having staff support enabled the bridging of transitional gaps, which is an ongoing and important issue (Parker et al. 2014). Like Fisher et al. (2018), most community site radiography professionals appraised and embraced the CS intervention. However, among the participants, some experienced smooth easing in and described the process as well organised (Beyers 2013; Ortiz 2016), whilst others experienced a steep climb (Eagle, Miles & Proeschold-Bell 2017) ‘to get on with it’.

Given the resource constraints experienced in terms of the ‘newcomers’ as gap fillers and dealing with sub-optimally functioning equipment, ‘tricky’ situations such as ‘no written protocols’ resulted in participants having to rely on their prior knowledge and experiential learning encounters to perform tasks in a meaningful way (Eagle et al. 2017). The outcomes were often a ‘waste’ of resources and tension with their CS placement supervisors (Laschinger et al. 2016); the novice professionals did experience instances of professional development, as well as growth and maturity, but also moments of critical reflection on career choice and fitness for the job. In addition, they had to learn to evaluate, make judgement calls, build relationships and apply self-regulation (Ortiz 2016; Sloane & Miller2017).

The strength of the study lies in providing insights into the ‘CS’ context in the radiography profession, in addition to the methodological aspect in capturing the continuum from pre-placement to post-placement, the proposed framework illustrates the transient stages in both their career and professional growth from student to the intermediate provisional professional to the professional practitioner. This framework could serve as a baseline for similar studies of this nature using the concept of professional learning.

## Limitations

There are several limitations and because of its qualitative nature, this study cannot be generalised. In addition, during follow-up interviews, only a small cohort was accessible mainly because of the unavailability of participants as a result of heavy work schedules and being placed at inaccessible CS placement centres with poor telephonic and cellular network reception. The use of a survey questionnaire should be considered, as well as including radiography academic training institutions from other provinces to assess preparedness of graduates as well as their respective community placement sites. With the latter, a mixed methods approach would be more appropriate. Also, consider a meta-study by including all the provinces. To get a holistic perspective, future studies could include members from the regulatory body as well as representatives of the National Department of Health to reach a common understanding and envisaged outcomes to enable smooth transition ‘in’ and ‘out’ of CS.

A collective review of the relevance of the CS placement intervention in the current health context and educational platforms should be undertaken by all stakeholders. Also, a feasibility study on the actual resources and sustaining of these resources to provide stability and a conducive environment, unlike the flux experienced in terms of inadequate resources and equipment breakdowns imposing constraints in service delivery, is indicated. One could also consider a preceptor programme.

From a radiography profession perspective, benchmark should be done internationally with an internship undertaking. This could be of benefit to inform by integration of best practice principles in preparation of these professionals prior to embarking on community placement.

## Conclusion

In conclusion, the value of CS cannot be disregarded, as there are gains that have been identified by these transitioning professionals. However, a review is inevitable to establish whether original intended goals, criteria and guidelines are still relevant. A shift from just a mere regulatory requirement and an awareness of the CS to one where the transition is seamless in benefiting the profession as well as the public in terms of access to quality health services is needed. All of this cannot be achieved through the disconnect between CS and a fit for work purpose approach.
